# Right-Sided Ectopic Intrathoracic Kidney Associated With Symptomatic Bochdalek Hernia in an Adult Indian Female: Case Report and Review of Literature

**DOI:** 10.7759/cureus.60598

**Published:** 2024-05-19

**Authors:** Satyanarayana Kummari, Rithika Ramadugu, Sameera Ramadugu, Mustafa Hussain Ansari, Saad Ali Ibrahim

**Affiliations:** 1 Radiodiagnosis, All India Institute of Medical Sciences, Nagpur, IND; 2 General Medicine, Kamineni Academy of Medical Sciences and Research Centre, Hyderabad, IND; 3 General Practice, Gandhi Medical College and Hospital, Hyderabad, IND; 4 General Practice, Shadan Institute of Medical Sciences, Hyderabad, IND

**Keywords:** elevated diaphragm, congenital diaphragmatic hernia (cdh), intrathoracic kidney, ectopic kidney, bochdalek hernia

## Abstract

Bochdalek hernia is an inherited posterior lateral defect in the diaphragm that allows the abdominal organs to herniate into the thoracic cavity. In addition to being the most prevalent variety of congenital diaphragmatic hernia (CDH), it is also the type that is observed on the left hemithorax the majority of the time. Ectopic kidney is an uncommon condition, and the occurrence of ectopic intrathoracic kidney is even more uncommon, accounting for only a few of all the cases of renal ectopias. The occurrence of intrathoracic kidney associated with Bochdalek hernia is infrequent among adult individuals and is typically an incidental finding. A 52-year-old obese female patient presented to the pulmonology outpatient unit and reported experiencing the symptoms of coughing, wheezing, and difficulty in breathing since three years. A chest radiograph revealed an elevated dome of the diaphragm on the right side. A computed tomography (CT) of the chest revealed a defect in the posterior aspect of the right hemi-diaphragm with herniation of the right kidney and retroperitoneal fat into the right hemi-thorax. CT urography showed normal size and enhancement of the intrathoracic kidney with prompt excretion of contrast into the pelvicalyceal system. With regard to the small size of the hernia and considering the absence of complications on CT urography, a conservative treatment was proposed to the patient. The patient was followed up every year. There was no occurrence of renal complications during the follow-up period. When evaluating patients with 'elevated hemi-diaphragm' or thoracic 'mass', it is essential to check for the presence of intrathoracic kidney to avoid undesirable surgical procedures and image-guided biopsies.

## Introduction

Bochdalek hernia is an inherited posterior lateral defect in the diaphragm that allows the abdominal organs to herniate into the thoracic cavity. This particular type of congenital diaphragmatic hernia affects around one in 2200 to 12,500 live births; it is more common on the left side of the thorax. Ectopic kidney is an uncommon condition that occurs in around one out of every 1000 live births. The occurrence of ectopic intrathoracic kidney is extremely uncommon, accounting for less than 5% of all renal ectopias [1-5]. The majority of these cases are observed in males in a ratio of 2:1 compared to females. It has been observed that the occurrence of intrathoracic renal ectopia due to congenital diaphragmatic hernia is less than 0.25%, with the majority of cases affecting the left side [1]. Right-sided Bochdalek hernia is an extremely uncommon occurrence, and the occurrence of right-sided intrathoracic kidney with Bochdalek hernia is much more uncommon. The presence of intrathoracic renal ectopia is commonly identified as an unintended observation on a chest radiograph, which mimics the appearance of a mass in the posterior mediastinum and necessitates additional assessment [6, 7]. In this case report, we describe an exceptional case of an adult Indian woman with symptomatic right-sided ectopic intrathoracic kidney with a Bochdalek hernia.

## Case presentation

**Figure 1:**
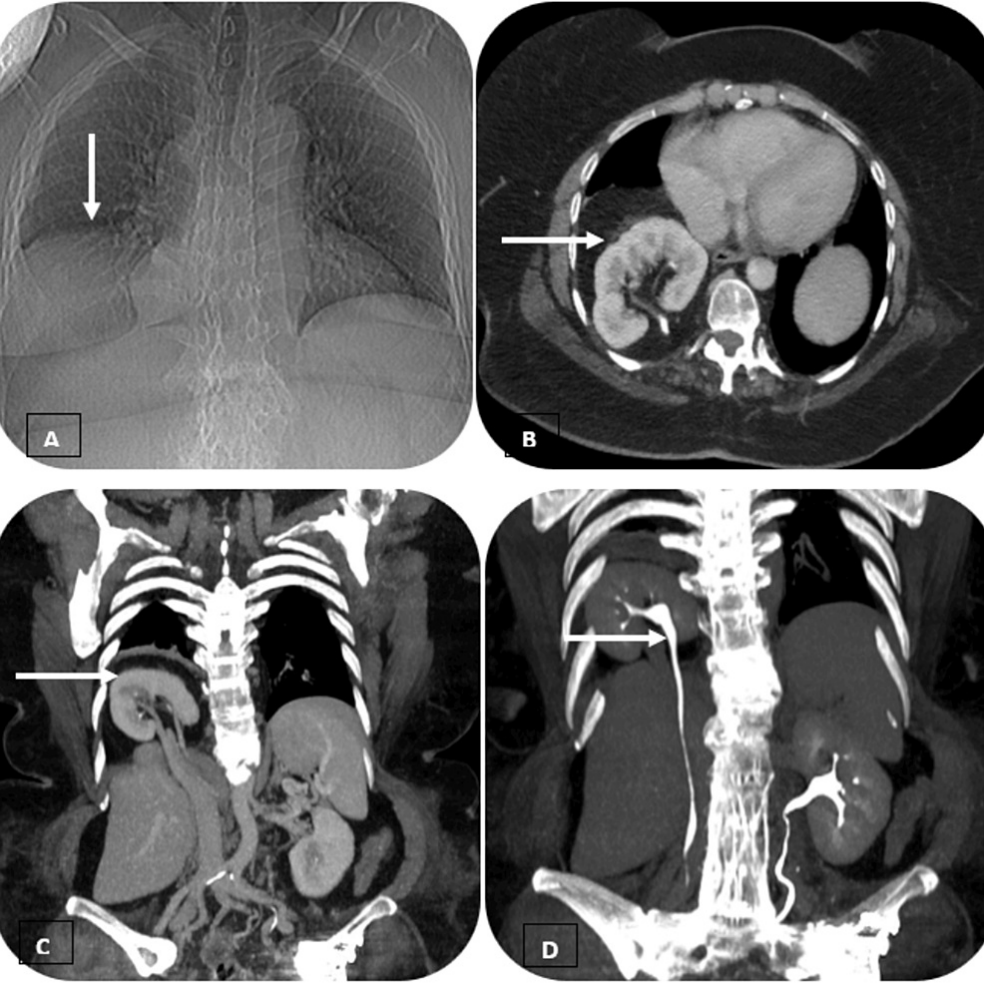
A: Radiograph of the chest showed elevated right dome of the diaphragm (white arrow); B (axial view) and C (coronal view): contrast-enhanced CT (CECT) of the chest and abdomen show normal size, attenuation and enhancement of the ectopic right intrathoracic kidney (white arrow); D: Coronal view of CT urography shows prompt excretion of contrast into the pelvicalyceal system of ectopic right intrathoracic kidney (white arrow).

A 52-year-old obese female patient presented to the pulmonology outpatient unit and reported experiencing the symptoms of coughing, wheezing, and difficulty in breathing since three years. The patient reported similar complaints intermittently over the past 10 years and also during her pregnancy, which was considered to be physiological by the treating physician. The patient reported no loss of appetite or weight. There was no history of fever, chest pain, or vomiting. Vital parameters were normal. On initial examination, chest auscultation revealed diminished breath sounds, dullness to percussion on the right lower zone, and bilateral rhonchi. Routine investigations like complete blood count (CBP), C-reactive protein (CRP), and absolute eosinophil count were done, which were all within the normal ranges. Sputum testing for *Mycobacterium tuberculosis* screening was negative. Chest radiograph was done at a local referring hospital, which showed elevated right dome of the diaphragm (Figure 1).

The patient was then referred to the Department of Radiodiagnosis for the computed tomography (CT) of the chest for further evaluation. The computed tomography of the chest revealed a defect in the posterior aspect of the right hemi-diaphragm with herniation of right kidney and retroperitoneal fat into the right hemi-thorax. The intrathoracic kidney was normal in size, contour, and attenuation. The renal pelvis was directed posteromedially (Figure 1). Atelectatic changes were noted in the basal segments of the right lower lobe. CT urography showed normal enhancement of the intrathoracic kidney with prompt excretion of contrast into the pelvicalyceal system (Figure 1). No other abnormalities were detected in the chest and abdomen. Considering the small size of the hernia and the absence of complications on CT urography, a conservative treatment was proposed to the patient. The patient was followed up every year with a thoraco-abdominal computed tomography and a biological evaluation of the renal function. There was no occurrence of renal complications during the follow-up period.

## Discussion

In 1848, Bochdalek was the first to describe the Bochdalek hernia, which is an inherited posterior lateral defect in the diaphragm that allows the abdominal organs to herniate into the thoracic cavity. It is the most frequent type of CDH, and it is caused by the failure of the pleuroperitoneal ducts to close at around eight weeks of gestation, as well as by primordial connections between the thoracic and abdominal cavities. It is typically observed in infants and children more frequently and is usually symptomatic in younger age group, presenting with gastrointestinal symptoms in infants and respiratory symptoms seen in children [6, 7]. It is often misdiagnosed as pulmonary tuberculosis, pneumothorax, or pleuritis in those presenting with respiratory symptoms. This condition can be diagnosed in antenatal stages and infants through ultrasonography and confirmed through a CT or MRI scan. It is rather uncommon in adulthood with less than 110 reported cases [8]. It is usually asymptomatic in adults and noted as an incidental finding in individuals undergoing evaluation prior to surgeries. The symptomatic cases present with chronic symptoms: gastrointestinal symptoms like recurrent pain abdomen, vomiting, and postprandial fullness; respiratory symptoms like coughing, wheezing, difficulty in breathing, and pain in the chest [8]. This defect is commonly seen on the left side (80%) as compared to the right side (20%). Bilateral Bochdalek hernias are very rare and often life-threatening. Left-sided Bochdalek hernias generally present with gastrointestinal symptoms. The organs that are most frequently herniated are the omentum, the colon, the spleen, the kidney, and the pancreas on the left side and the liver on the right side. In very rare cases, ectopic intrathoracic kidney is reported to occur due to Bochdalek hernia [9].

Ectopic kidney is an uncommon condition that occurs in around one out of every 1000 live births. The occurrence of ectopic intrathoracic kidney is even more an infrequent phenomenon, accounting for fewer than 5% of all cases of renal ectopias. This condition is prevalent among males and manifests on the left side in around 80-90% of cases. The incidence of Bochdalek hernia associated with intrathoracic kidney is reported to be <0.25% [1] with most of the cases concerning the left side. The incidence of right-sided Bochdalek hernia is rare with 32 cases reported in medical literature so far and concomitant, right-sided intrathoracic kidney and Bochdalek hernia is even rarer with only two cases reported in adults [1, 9], three cases in the children [10-12], and two prenatal cases thus far [13, 14]. Through the foramen of Bochdalek, the kidney exits the retroperitoneal space and enters the thoracic cavity but not the pleural space. This particular patient did not exhibit any additional abnormalities in the chest and abdomen.

A posterior-facing hilum, a long ureter, a more proximal origin of the blood vessels of the kidney and at times medial deviation of the lower pole of the kidney are some of the anatomical features that are derived from the developmental rotation of the ectopic intrathoracic kidney [15]. Even though these abnormalities are present, the intrathoracic kidney is typically in full-functioning condition. In contrast to other intrathoracic renal ectopias, the intrathoracic kidney that is associated with Bochdalek hernia is moveable and can be lowered from the chest into the retroperitoneal region. The majority of the kidneys located within the thoracic cavity continue to be asymptomatic and surgery is only necessary in case of ureteral obstruction or vesicoureteral reflux. Asymptomatic patients are managed conservatively and surgical management is preferred in symptomatic or complicated cases. The intrathoracic kidney is reduced, and the defect is closed by tension-free wire, non-absorbable suture or using a prosthetic patch for larger defects [15].

## Conclusions

Bochdalek hernia is an inherited posterior lateral defect in the diaphragm that allows the abdominal organs to herniate into the thoracic cavity. In the clinical setting, intrathoracic kidneys are uncommon medical conditions that present clinicians with a number of diagnostic and treatment challenges. The occurrence of intrathoracic kidney associated with Bochdalek hernia is uncommon, with most of the cases concerning the left side. Right-sided intra-thoracic kidney with Bochdalek hernia is much more uncommon. In most cases of ectopic thoracic kidney, its structure and function are normal; despite this, it should be considered in the evaluation of patients with ‘elevated hemi diaphragm’ or thoracic 'mass’ to prevent unnecessary surgical interventions and image-guided biopsies.
